# A multimodality localization technique for radio-guided surgery

**DOI:** 10.1186/1477-7819-5-43

**Published:** 2007-04-25

**Authors:** Seza A Gulec, Erica Hoenie, Kristan Rheinheimer

**Affiliations:** 1 Goshen cancer Institute at Goshen Health System, Goshen, IN, USA

## Abstract

**Background:**

Intraoperative localization of image or endoscopy-detected lesions occasionally pose surgical challenges due to the small lesion size and/or difficult anatomic exposure. Identification of such lesions can be facilitated using a hand-held gamma probe with utilization of Tc-99m macroaggregate albumen (MAA) localization technique. The radiopharmaceutical injection can be performed using ultrasound (US) or endoscopy guidance.

**Case presentations:**

The clinical use of the Tc-99m MAA protocol gamma probe-guided surgery was discussed in three representative cases. Surgical indication was diagnostic exploration in two patients with suspicious lymphadenopathy, and determination of extent of surgical resection in a patient with polyposis. Lesion localization with 100 microcurie (3.7 MBq) Tc-99m MAA prior to surgical exploration resulted in definitive localization of lesions intraoperatively.

**Conclusion:**

The use Tc-99m MAA deposition technique at the site of surgical target is a highly efficient radio-guided surgery technique with definitive impact on the success of surgical exploration in selected indications.

## Background

The role of gamma probes in surgical oncology practice has been well established [1-9]. Surgical performance with intraoperative gamma probe detection is critically dependent on target to surrounding background ratio (TBR). This ratio, for localization techniques that involve systemic administration of radiopharmaceuticals, is a function of radiopharmaceutical uptake and clearance kinetics. Probe's ability to discern the target signal also is a major technical factor in the clinical success. A minimum TBR of 1.5:1 is needed in the operative field for the operating surgeon to be comfortable that the differences between the target tissue and normal adjacent tissue are real [10]. Obtaining a satisfactory TBR is always a significant technical challenge with localization techniques using systemic administration of radiopharmaceuticals. Administration of a locally-entrapped radiopharmaceutical in or around the target tissue results in an ideal TBR.

## Case presentations

### Case 1

**Figure 1:**
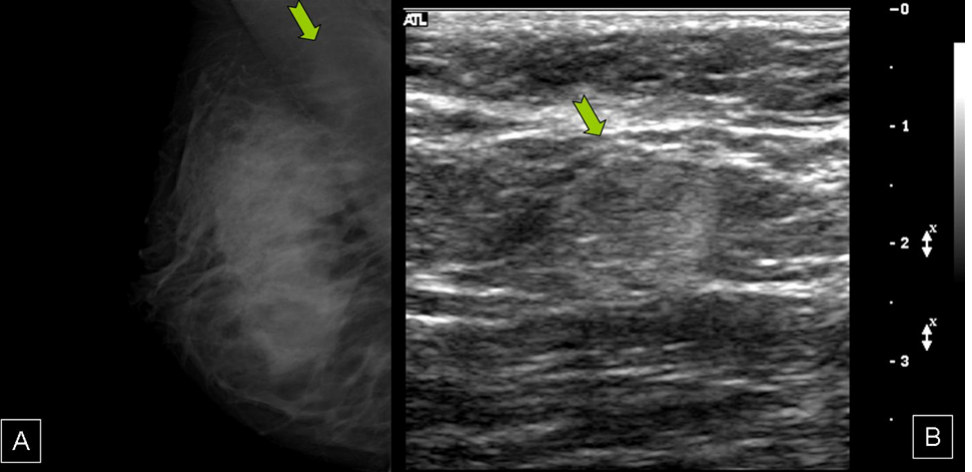
a) Right axillary lymphadenopathy demonstrated on mammogram. b) Ultrasonogram image. The node measures 1 cm and is not clinically palpable.

A 32 year-old woman with a history of T2-N1 left breast cancer, diagnosed 2 years ago and treated with mastectomy-immediate reconstruction, presented with a right axillary lymphadenopathy. Upon clinical exam, the lesion was non-palpable and measured approximately 1 cm by US. The lymphadenopathy persisted on a follow-up; FDG-PET imaging was negative. An US-guided FNA was non-definitive without evidence of malignancy. An increase in size of the node was noted at the subsequent follow-up. A clinical decision was made for an excision biopsy. The node was injected with Tc-99m macroaggregate albumen (MAA) and lymphazurin blue 2 h prior to the planned operation. At surgery, the hot-spot was readily identified. Probe localization was distinctly focal over a level-I node, which was accessed with minimal dissection. Blue dye facilitated surgical exposure and dissection. Surgical pathology revealed chronic inflammatory changes (Figure 1).

### Case 2

**Figure 2:**
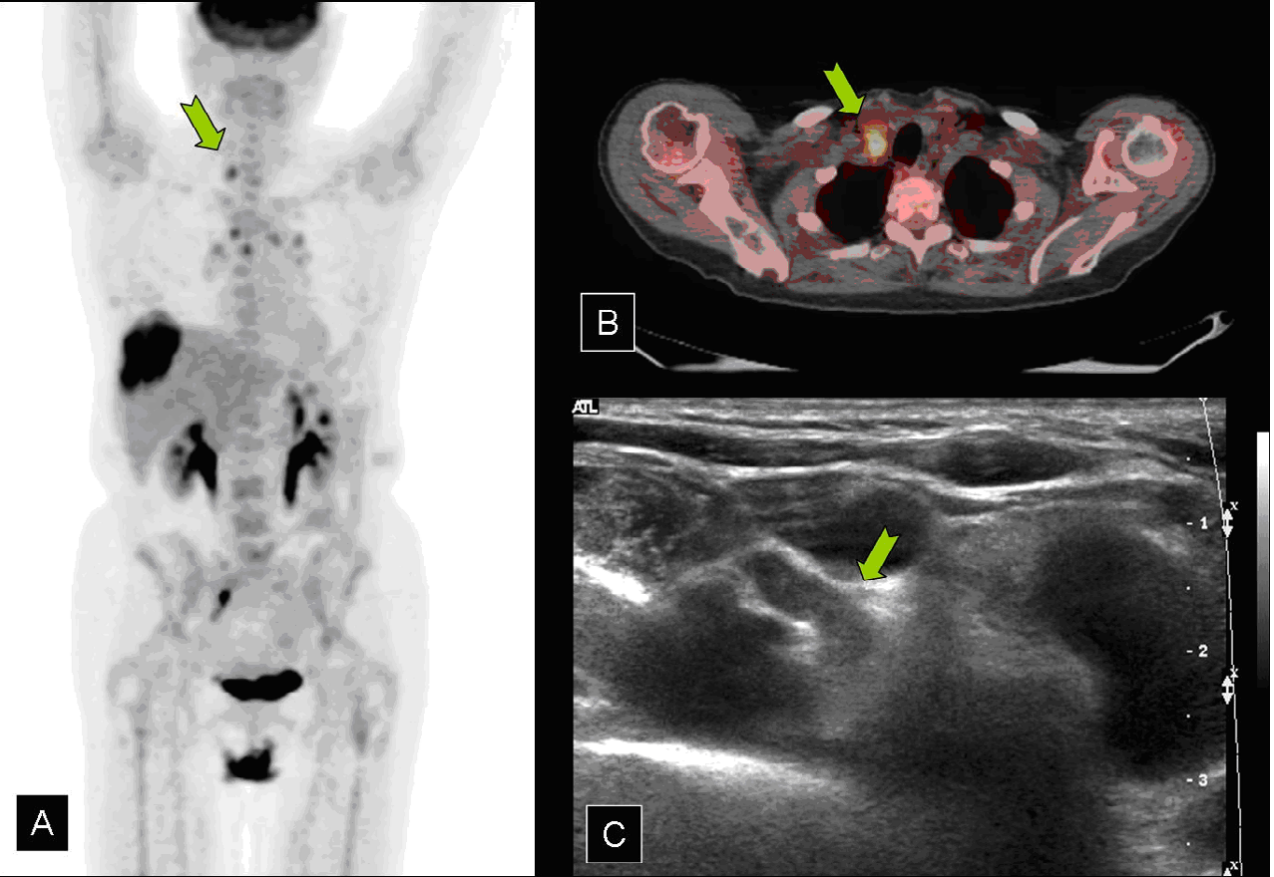
a) Retroclavicular lymph node demonstrated on composite PET image. b) PET/CT demonstrating large liver lesion. c) Ultrasound of lesion.

A 65 year-old woman with history of colorectal cancer (CRC) and non-Hodgkin's lymphoma of the scalp, presented with mediastinal and retroclavicular lymphadenopathy. CRC was diagnosed 2 years ago, and was treated with R-colon resection. Scalp lymphoma was diagnosed 6 months ago, when the patient presented with a 15 cm scalp lesion. Staging work-up at that time revealed positive FDG uptake in the scalp lesion (SUV:18), multiple mediastinal and R-retroclavicular lymph node (SUV:3.2), and a 12 cm liver lesion (SUV: 8.5). Biopsy of the liver lesion was consistent with the colon primary. Nodal findings were concluded to indicate a stage III lymphoma, and a sequential intensity-modulated radiation therapy (IMRT) and chemotherapy using CHOP regimen was administered. There was a complete clinical and FDG-PET/CT response in the scalp lesion. Post-treatment FDG-PET/CT showed persistence of mediastinal and R-retroclavicular nodal uptake. The R-retroclavicular node was injected with Tc-99m MAA and lymphazurin blue 2 h prior to the planned operation. At surgery, the hot-spot was readily identified. Probe localization was distinctly focal over an internal jugular-innominate vein confluence lymph node. The target was accessed through an incision made over the hot-spot. The line-of-sight provided a safe surgical dissection. The blue dye facilitated surgical exposure and dissection. Surgical pathology revealed a chronic granulomatous disease. The patient was restaged to have a stage I scalp lymphoma, and remained in complete remission following treatment. Stage IV CRC was also concluded to be a liver-only disease, which allowed her to be considered for liver-directed therapy (Figure 2).

### Case 3

**Figure 3:**
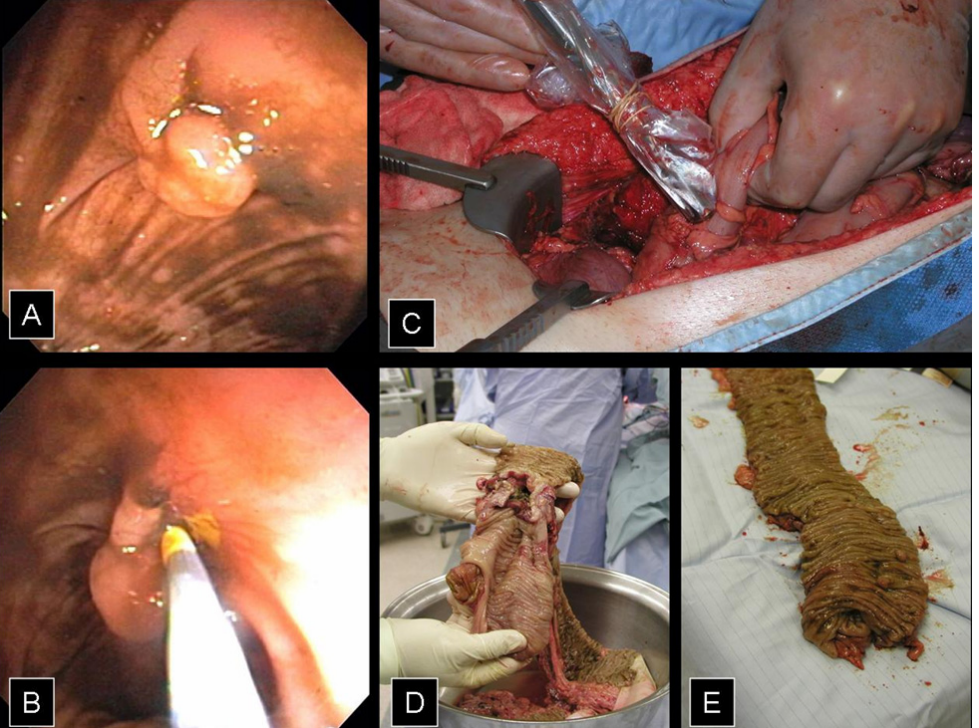
A and B) Sigmoid colon polyp and endoscopic injection of the base. C) Intaoperative gamma probe localization of Tc-99m MAA injection site. D and E) Distribution of multiple polyps in the resected specimen.

A 69 year-old man presented with anemia, subsequent colonoscopy revealed a right colon cancer and multiple polyps throughout the colon. Severe dysplastic changes were noted in a sessile polyp in the transverse colon and in a pedunculated polyp in the sigmoid colon of villous type. The descending colon polyp was completely excised colonoscopically and the site was submucosally injected with Tc-99m MAA and lymphazurin blue 18 h prior to a planned operation. At surgery, the ascending colon lesion was readily identified. None of the polyps were palpable. A slight, relatively diffuse discoloration of submucosal blue dye was noted at the site of injection. Probe localization was distinctly focal. A total abdominal colectomy was performed with 2-cm margins distal to the focal Tc-99m MAA signal. Surgical pathology confirmed complete resection with free margins (Figure 3).

### Gamma probe-guided surgery protocol

The patients receive an injection of 0.1 mCi (0.037 MBq) (in 0.1 ml solution) Tc-99m MAA the morning of planned surgery. Lymph node injections are given most conveniently under US. For colon polyp localization, the injection using the same activity and volume is given endoscopically. Surgical exploration is scheduled within 6 hours post injection of the radiopharmaceutical. The injected activity might be doubled if surgery is planned 6–12 hours after the injection (Table 1). Standard gamma probe settings (photopeak of 140 keV, Window of 20%, and a threshold of 136 keV) and operative technique are used in the operating room.

**Table 1:** Gamma probe-guided surgery protocol for Tc-99m MAA

**Tc-99m MAA Lesion Localization with image/endoscopy guidance**	
Radiopharmaceutical Dose/Administration	• Tc-99m MAA • 0.1 mCi (.0037 MBq)/direct injection
Standard Imaging Protocol	• No nuclear medicine imaging needed
Timing of Surgical Exploration	• Within 6 h post-injection
Patient Preparation	• Not required
Gamma Probe	• Standard medium-energy gamma probe
System set-up	• Analyzer Settings: Photopeak: 140 keV, Window: 20%, Threshold: 136 keV (In commercial systems this is a default setting) • Verify calibration and settings of the system • Cover the probe with sterile plastic sleeve
Intra-operative Use	• Probe survey at *counts- per-second *mode (Dynamic pitch range feed-back helpful) • Hot-spot confirmation with TBR>1.5 at *10-second count *mode (TBR ratio feed-back helpful) Avoid simultaneous electrocautery use

## Discussion

The use Tc-99m MAA deposition technique at the site of surgical target is a highly efficient radio-guided surgery technique with definitive impact on the success of surgical exploration in selected indications. Administration of the locally-entrapped Tc-99m MAA in or around the target tissue results in an ideal TBR. Tc-99m MAA, when injected into the tissues, remains almost stationary with a minimal local diffusion. Particle degradation, slow lymphatic absorption and phagocytosis constitute the principal mechanisms for MAA clearance. The biologic half life of MAA is approximately 6 hours. The effective half life of Tc-99m MAA is calculated at approximately 3 hours when the 6-hour physical half life of Tc-99m is factored in (1/2 T_Eff _= 1/2 T_Biol _+ 1/2 T_Phys_). A 0.1 mCi Tc-99m provides satisfactory signal intensity for gamma probe detection within a wide range of time frame. Surgical procedure can be scheduled any time after injection up to 6 hours. (Suggestion: Surgical procedures can be scheduled any time during the 6 hours post injection.) Tc-99m MAA injection can be given under CT, US or endoscopic guidance. Lymphazurin blue (blue dye) can be added to the injectate to facilitate dissection by providing a visual aid.

## Conclusion

Major applications of the technique include localization of lymph nodes and colonic polyps. The technique may also be used in localization of non-palpable breast lesions as an adjunct to needle/wire localization techniques.

## Competing interests

The author(s) declare that they have no competing interests.

## Authors' contributions

**SG **– Design, Acquisition, analysis and interpretation of data, Drafting manuscript, Critical revision

**EH **– Analysis of data, Editing of the manuscript

**KR **– Acquisition of data
